# Electro-Acupuncture Effects Measured by Functional Magnetic Resonance Imaging—A Systematic Review of Randomized Clinical Trials

**DOI:** 10.3390/healthcare12010002

**Published:** 2023-12-19

**Authors:** Jorge Magalhães Rodrigues, Cristina Ventura, Manuela Abreu, Catarina Santos, Joana Monte, Jorge Pereira Machado, Rosa Vilares Santos

**Affiliations:** 1 IPTC—Research Department in Complementary Therapies, Portuguese Institute of Taiji and Qigong, 4470-765 Maia, Portugal; 2 ABS—Health Level Department, Atlântico Business School, 4405-604 Vila Nova de Gaia, Portugal; 3 CBSin—Center of BioSciences in Integrative Health, 4200-135 Porto, Portugal; 4 ICBAS—School of Medicine and Biomedical Sciences, University of Porto, 4050-313 Porto, Portugal; 5 FMUP—Faculty of Medicine, University of Porto, 4200-319 Porto, Portugal

**Keywords:** electro-acupuncture, fMRI, neurosciences, complementary therapies, integrative medicine

## Abstract

Introduction: Electro-acupuncture, an innovative adaptation of traditional acupuncture, combines electrical stimulation with acupuncture needles to enhance therapeutic effects. While acupuncture is widely used, its biological mechanisms remain incompletely understood. Recent research has explored the neurophysiological aspects of acupuncture, particularly through functional magnetic resonance imaging (fMRI) to investigate its effects on brain activity. Methods: In this systematic review, we conducted an extensive search for randomized clinical trials examining electro-acupuncture effects measured by fMRI. We employed strict eligibility criteria, quality assessment, and data extraction. Results: Five studies met our inclusion criteria and were analyzed. The selected studies investigated electro-acupuncture in various medical conditions, including carpal tunnel syndrome, fibromyalgia, Crohn’s disease, irritable bowel syndrome, and obesity. Notably, electro-acupuncture was found to modulate brain activity and connectivity in regions associated with pain perception, emotional regulation, and cognitive processing. These findings align with the holistic approach of traditional Chinese medicine, emphasizing the interconnectedness of body and mind. Discussion: In carpal tunnel syndrome, electro-acupuncture at both local and distal sites showed neurophysiological improvements, suggesting distinct neuroplasticity mechanisms. In fibromyalgia, somatosensory electro-acupuncture correlated with reduced pain severity, enhanced brain connectivity, and increased gamma-aminobutyric acid levels. For Crohn’s disease, electro-acupuncture influenced the homeostatic afferent processing network, potentially mitigating gut inflammation. Electro-acupuncture for irritable bowel syndrome led to decreased activity in the anterior cingulate cortex, offering pain relief, while electro-acupuncture for obesity impacted brain regions associated with dietary inhibition and emotional regulation. Conclusion: This systematic review provides evidence that electro-acupuncture can positively impact a range of medical conditions, possibly by modulating brain activity and connectivity. While the quality of the reviewed studies is generally good, further research with larger sample sizes and longer-term assessments is needed to better understand the mechanisms and optimize electro-acupuncture protocols for specific health conditions. The limited number of studies in this review emphasizes the need for broader investigations in this promising field. The research protocol was registered in PROSPERO (CRD42023465866).

## 1. Introduction

Electro-acupuncture is a form of acupuncture, a traditional Chinese medical practice, in which electrical stimulation is added to the acupuncture needles that are inserted into specific points on the body [1]. This technique combines the principles of traditional acupuncture with modern technology to enhance the therapeutic effects of the treatment.

Despite the use of acupuncture being widespread around the world, its biological action mechanisms are still not fully understood.

Nakatani [2], for example, proposed that acupuncture points and body “meridians” were a consequence of the visceral-cutaneous reflex, hypothesizing why acupuncture might work, specifically the connection between the skin and the internal organs. In fact, Fang et al. [3] studied the axon bifurcation in primary C-nociceptive neurons that innervate both the skin and a visceral organ and suggested that cutaneous hypersensitivity on certain locations of the body surface might serve as an indicator of pathological conditions in the corresponding visceral organ. Additionally, the back Shu point system commonly used in acupuncture might be explained by the viscerosomatic reflex [4,5] which is the result of the effect of afferent stimuli arising from a visceral disorder on the somatic tissues. According to Beal [6], the reflex action starts with the visceral receptors sending afferent impulses to the dorsal horn of the spinal cord, where they connect with interconnecting neurons. The interconnecting neurons carry the stimuli to the sympathetic and peripheral motor efferents, ultimately leading to sensory and motor alterations in the skeletal muscle, viscera, blood vessels, and skin tissues.

Nevertheless, from a neurophysiological perspective, acupuncture can be seen as a form of intricate somatosensory stimulation [7].

Since the mid-1990s, there has been a growing interest in using imaging techniques to study the mechanisms of acupuncture [8,9]. Positron emission tomography (PET), single photon emission computed tomography (SPECT), and magnetic resonance imaging (MRI) have all been employed, and there is also a growing interest in using electroencephalography (EEG). However, functional magnetic resonance imaging (fMRI) has become the dominant method in the field of brain mapping due to its minimal invasiveness, lack of radiation exposure, excellent spatial resolution, and relative ease of access [10]. With the evolution of assessment techniques, it has become apparent that the brain’s response to acupuncture stimuli involves a wide network of regions that are consistent not only with somatosensory processing but also with affective and cognitive processing [10].

In particular, fMRI studies have revealed that acupuncture can modulate brain activity and connectivity in areas associated with pain perception, emotion regulation, and cognitive processing [11,12,13]. These findings align with the holistic approach of acupuncture in traditional Chinese medicine, where the body and mind are considered interconnected.

By applying fMRI and other advanced imaging techniques, researchers are gradually unravelling the complex neurophysiological mechanisms behind acupuncture’s therapeutic effects. This interdisciplinary approach bridges the ancient practice of acupuncture with modern neuroscience, bringing us closer to a comprehensive understanding of how acupuncture influences the human body and mind.

This systematic review, based on recent randomized clinical trials (RCTs), seeks to offer insights into the potential applications of electro-acupuncture and elucidate underlying brain mechanisms, particularly through the analysis of fMRI results.

Through this research, we seek to enhance the understanding of its clinical efficacy in addressing various medical conditions, thereby contributing valuable evidence for its integration into the healthcare sector as part of the integrative medicine concept.

## 2. Methods

### 2.1. Search Strategy

The researchers conducted a thorough search for RCTs that examined the effects of electro-acupuncture using fMRI as a measurement tool. Multiple databases were searched, including PubMed, The Cochrane Library, Web of Science, Science Direct, and Scielo, from 2017 until 13 February 2022. The search strategy used the following formula: ((electroacupuncture) OR (electro-acupuncture)) AND ((fMRI) OR (functional magnetic resonance imaging)). The results were then filtered by randomized clinical trials published in the last 5 years. 

The resulting list of studies was inserted into a web app called Rayyan [14], where duplicates were removed, and three researchers independently reviewed the title and abstract of each study to determine its adequacy. Any disagreements were resolved through discussion until a unanimous decision was reached.

After retrieving the full text of the studies, the same process was applied, and a quality assessment was performed. The research protocol was registered with the international prospective register of systematic reviews (PROSPERO) under the record number CRD42023465866. 

### 2.2. Eligibility Criteria

Only RCTs conducted on human subjects and reported in the English, Portuguese, Spanish, or French languages were considered. The reasons for restricting our search to RCTs was due to their inherent advantages, including the ability to minimize bias through randomization, establish causal relationships, ensure controlled conditions for interventions, achieve higher statistical power, uphold ethical standards in participant recruitment, and contribute to the robustness of our findings, thus prioritizing the reliability and internal validity of our research.

Studies involving animal subjects or those that studied traditional acupuncture or laser acupuncture were excluded. Additionally, only high-quality articles were included, whereas studies with low or moderate quality were excluded.

### 2.3. Quality Assessment

To ensure that only high-quality articles were included in this study, a quality assessment was conducted during the article selection phase. For this purpose, we used the modified Downs and Black [15] checklist, which is designed to assess the methodological quality of randomized studies of health care interventions. This checklist includes 27 questions and allows for a maximum possible score of 28. Scores of 26 to 28 are considered “excellent”, scores of 20 to 25 are considered “good”, scores of 15 to 19 are considered “fair”, and scores of 14 or below are considered “poor”. By applying this checklist, we were able to fulfill our exclusion criteria and select only articles that met our standards for good and excellent quality.

### 2.4. Data Extraction

Data extraction was performed by two investigators, and any discrepancies were resolved through discussion with a third investigator to reach a unanimous decision. For each eligible article, we recorded the author(s) name(s), year of publication, title, and journal where it was published, as well as information on the sample, condition, groups, and participants who completed the study, acupuncture technique used, duration and frequency of the intervention, outcome measures, fMRI analysis/task, and main results.

## 3. Results

**Figure 1:**
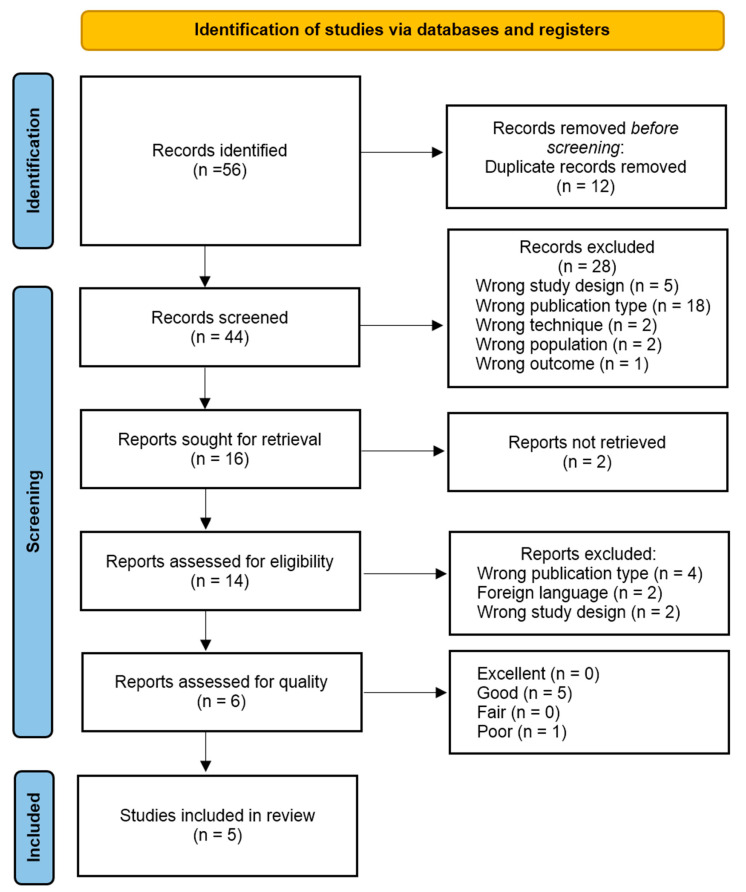
Preferred Reporting Items for Systematic Reviews and Meta-Analyses (PRISMA) flowchart.

Figure 1 displays the flowchart from the Preferred Reporting Items for Systematic Reviews and Meta-Analyses (PRISMA) developed by Page et al. [16] that was used for the study selection in this systematic review.

The initial database search yielded 56 records, which were reduced to 44 after eliminating duplicates. Following the title and abstract screening, 16 studies were considered for retrieval, with only 2 studies not being retrieved. A full-text review and quality assessment were conducted on 14 studies, and eventually, 5 studies were included in the final analysis. Figure 1 reports additional information and reasons for exclusion.

The included studies investigated electro-acupuncture effects measured by fMRI on a small variety of conditions.

One article studied the electro-acupuncture effect on carpal tunnel syndrome [17], one did so on fibromyalgia [18], one did so on Crohn’s disease [19], one did so on irritable bowel syndrome [20], and one did so on obesity [21].

Two studies [19,20] compared the use of electro-acupuncture to moxibustion, two compared electro-acupuncture to sham acupuncture [17,21], and one compared electro-acupuncture to mock laser acupuncture [18].

The intervention’s session duration was 30 min in three studies [19,20,21], 25 min in one study [18], and 20 min in one study as well [17].

The average treatment duration was 7.6 weeks amongst the included studies, with a weekly frequency of 3 sessions in 2 studies [19,21], 2 sessions in two other studies [17,18], and 6 sessions in another [20].

Regarding the quality assessment, all included studies scored “good”, with none scoring “excellent”.

The summary of the studies is shown in Table 1.

## 4. Discussion

### 4.1. Local vs. Distal Electro-Acupuncture for Carpal Tunnel Syndrome

The study of Maeda, Kim, Kettner, Kim, Cina, Malatesta, Gerber, McManus, Ong-Sutherland, Mezzacappa, Libby, Mawla, Morse, Kaptchuk, Audette and Napadow [17] employed a three intervention-arm design for the treatment of carpal tunnel syndrome by dividing the 80 participants by (1) verum electro-acupuncture applied locally to the affected hand; (2) verum electro-acupuncture at distant body sites near the ankle and contralateral wrist; and (3) local sham electro-acupuncture using placebo needles.

All acupuncture interventions led to reduced symptom severity, but verum acupuncture (both local and distal) was more effective than sham acupuncture in improving neurophysiological outcomes.

This was observed in both wrist-localized measurements (e.g., median sensory nerve conduction latency) and brain measurements (e.g., separation distance between cortical areas for digits 2 and 3). Notably, improvements in cortical separation distance for digit 2 and 3 correlated with sustained symptom improvement at the 3-month follow-up.

The study also used diffusion tensor imaging to analyze white matter microstructure near S1. Patients with carpal tunnel syndrome had elevated fractional anisotropy in specific regions compared to healthy adults. Improvement in median nerve latency was associated with decreased fractional anisotropy in distinct regions: (1) contralateral hand area after verum acupuncture only; (2) ipsilateral hand area after local acupuncture only; and (3) ipsilateral leg area after distal acupuncture only. These findings indicate that local and distal acupuncture stimulates different somatosensory cortex subregions, leading to region-specific improvements in median nerve function.

The study suggests that both local and distal acupuncture can effectively alleviate carpal tunnel syndrome symptoms. The improvements observed in neurophysiological outcomes are likely attributed to distinct neuroplasticity mechanisms in S1. These results are also supported by the study of Chen et al. [22] which found that the combination of both local and distal acupuncture resulted in improved therapeutic benefits for carpal tunnel syndrome, notably surpassing the efficacy of local acupuncture only. Since there are different neuroplasticity mechanisms that may contribute to improved outcomes, it seems plausible that combining mechanisms may result in superior benefits. Moreover, it has been hypothesized that the S1 cortex plays a role in pinpointing and distinguishing pain, directly receiving pain signals via the ascending nociceptive pathway. Moreover, chronic pain has been linked to both irregular functional activity and structural alterations within the S1 cortex [23,24]. In light of this, it is conceivable that the S1 cortex tends to activate in response to pain, and the therapeutic effectiveness of contralateral distal acupuncture may stem from its capacity to counteract this activation.

On the contrary, in the context of Yan et al.’s study [25], it was noted that ipsilateral distal electro-acupuncture led to a reduction in degree centrality within the bilateral cerebellum and the right thalamus. Traditionally, the cerebellum has primarily been associated with motor functions. Nevertheless, recent research suggests that it assumes a role in a range of integrative functions and neural processes that extend beyond the domain of motor control, particularly in the context of pain processing [26,27,28,29,30,31,32].

Furthermore, changes in somatotopy within the cortex can predict long-term clinical outcomes for carpal tunnel syndrome patients, which is also supported in the study by Kim et al. [33].

Other previous studies also showed positive results in the use of acupuncture for carpal tunnel syndrome [34,35,36,37].

### 4.2. Electro-Acupuncture for Fibromyalgia

Our review identified one study [18] that aimed to determine the specific influence of somatosensory input on fibromyalgia pain relief through acupuncture and the related brain circuits. Seventy-six patients were divided into a group receiving electro-acupuncture with somatosensory input and the other receiving mock laser acupuncture with no somatosensory input. Pain severity levels were measured and functional brain network connectivity was assessed using resting state fMRI imaging and proton magnetic resonance spectroscopy in the right anterior insula before and after treatment.

Patients who received electro-acupuncture demonstrated a greater reduction in pain severity compared to those who received mock laser acupuncture. Electro-acupuncture recipients exhibited increased connectivity between the leg’s S1 and the anterior insula. This enhanced connectivity was associated with reduced pain severity and increased levels of γ-aminobutyric acid (GABA+) in the anterior insula following electro-acupuncture therapy. Additionally, higher GABA+ levels in the anterior insula correlated with lower pain severity. Importantly, the changes in GABA+ levels mediated the relationship between alterations in S1 leg and the anterior insula connectivity and pain severity reduction.

Overall, the study found that the somatosensory element of electro-acupuncture influences primary somatosensory connectivity linked with insular neurochemistry. This modulation contributes to a decrease in pain severity in fibromyalgia patients.

GABAergic inhibition seems to be engaged in the anterior insula to alleviate nocifensive behavior [38]. The findings of the study suggest that S1 leg might be communicating with the anterior insula to mitigate clinical pain through GABAergic inhibition. Otherwise, acupuncture could transiently enhance pronociceptive signaling between the S1 leg subregion and the anterior insula, potentially triggering the activation of endogenous descending inhibitory systems that counteract this effect through GABAergic inhibition of the anterior insula [18]. This process could resemble healing responses initiated by temporary injury [39].

In fact, a decrease in anterior insula GABA levels has been noted in fibromyalgia patients [40] along with a compensatory up-regulation of GABA type A receptors [41]. According to that, other interventions that enhance GABAergic neurotransmission have shown effectiveness for fibromyalgia treatment [42,43,44,45].

It is also important to note that the anterior insula is known to be hyperactive in fibromyalgia patients [46,47]. Patients who experience a post-therapy increase in anterior insula GABA+ levels might encounter a reduction in hyperactivity within the anterior insula, ultimately leading to analgesic effects [18]. The study of Li et al. [48] also supports the possibility that microglia and GABAA receptors might be involved in electro-acupuncture analgesia.

The positive results found in this article are in agreement with scientific literature on the research topic [49,50]. Acupuncture’s effect on fibromyalgia has increasingly been studied in the last decades and much interest has been gathered around electro-acupuncture [51].

### 4.3. Electro-Acupuncture on ST25, CV6, and CV12 for Crohn’s Disease

Bao, Wang, Liu, Shi, Jin, Wu, Zeng, Zhang, Liu and Wu [19] studied the effects of electro-acupuncture and moxibustion on bilateral ST25, CV6, and CV12 on brain connectivity in remission patients with Crohn’s disease.

In this study, both techniques significantly improved the outcome measures used to assess the effects of the interventions. However, it was concluded that the therapeutic effects of electro-acupuncture and moxibustion may involve different mechanisms.

In electro-acupuncture, the resting-state functional-connectivity (rsFC) values between the bilateral hippocampus, the anterior middle cingulate cortex (MCC), and the insula were significantly increased and supported by a negative correlation with the Crohn’s Disease Activity Index (CDAI) scores.

Other studies by the same first author showed that Crohn’s disease remission patients display abnormalities in the homeostatic afferent processing network [52,53], to which the MCC and insula correspond. This network processes the interoceptive input [54,55,56] as a regulation of visceral sensation, homeostasis, emotion, and pain. Body and visceral painful and non-painful stimulation may activate this network [55,57]. Moreover, the insular cortex, highly related to interoception [58], may be able to detect inflammation in the gut [59]. The effect of electro-acupuncture in the MCC and the insula possibly result in a modulation in gut inflammation via improvement of the rsFC values of the homeostatic afferent processing network.

The increased rsFC values with the hippocampus also suggest that electro-acupuncture may improve cerebral integration via vagus nerve-mediated gastrointestinal signals.

These results are also supported by studies that observed benefits of applying acupuncture on Crohn’s disease [60,61,62] with possible positive effects on visceral pain [63], alleviating symptoms of visceral hypersensitivity and protecting the gastrointestinal tract [64] and assisting in the management of associated anxiety and depression symptoms [65,66].

### 4.4. Electro-Acupuncture on ST25 and ST37 for Irritable Bowel Syndrome

Zhao, Lu, Yin, Wu, Bao, Chen, Chen, Tang, Jin, Wu and Shi [20] conducted a trial to compare electro-acupuncture and moxibustion in patients with constipation-predominant irritable bowel syndrome.

By using bilateral Tianshu (ST25) and Shangjuxu (ST37) acupuncture points, the authors observed that both techniques were therapeutically effective in relieving abdominal pain, bloating, and abdominal discomfort. However, electro-acupuncture was more effective in improving defecation frequency, difficulty in defecation, and constipation, as well as relieving depression, anxiety, and other psychological symptoms.

Through the use of technologies like fMRI, researchers have identified distinct patterns of brain activity in individuals with certain medical conditions compared to those in healthy subjects. Using healthy controls as a pre-treatment comparison, this study further provided evidence about this association between visceral hypersensitivity and the central nervous system, confirming the abnormal brain-gut function in irritable bowel syndrome.

Regarding irritable bowel syndrome patients, the anterior cingulate cortex, the insular cortex, and the prefrontal cortex may show increased or decreased activation [67,68,69].

With electro-acupuncture, patients exhibited significantly decreased activity in the anterior cingulate cortex area after treatment. In this context, it is important to understand that the anterior cingulate cortex is related to pain perception and chronic pain regulation [70,71] and the results of the study suggest that electro-acupuncture therapy may be effective in improving pain signals.

Furthermore, constipation-predominant irritable bowel syndrome patients in this study exhibited increased activation of the insular cortex. As we have discussed before, the insula is part of the homeostatic afferent processing network [52,53] and is related to interoception [58]. Therefore, as it is involved in the regulation of visceral sensation, homeostasis, emotion, and pain, regulating this brain area may assist in managing irritable bowel syndrome symptoms.

Additionally, before treatment, constipation-predominant irritable bowel syndrome patients displayed more activity in the prefrontal cortex. The authors suggest that this might be because the signals from the anterior cingulate cortex and insular cortex were stimulating the prefrontal cortex more in these patients. In this study, a significant decrease in the activity of the prefrontal cortex was observed after electro-acupuncture treatment.

Clinical trials involving irritable bowel disease patients and the application of electro-acupuncture support the results of this study by showing promising application in comparison to medication [72], by down-regulating the abnormally increased colonic mucosa-associated neuropeptide substance P and vasoactive intestinal peptide expression [73], and by also improving abdominal pain and abdominal bloating [74].

### 4.5. Electro-Acupuncture for Obesity

Ren, Xu, von Deneen, He, Li, Zheng, Zhang, Li, Han, Cui, Ji, Nie and Zhang [21] studied the acute and long-term effects of electro-acupuncture on brain activity and rsFC in obese patients. As a control, a sham-acupuncture group was used for comparison, but the discussion of those results is not within the scope of this review. However, creating a placebo control for practices like acupuncture can be challenging, and it is important to adapt methods and evaluations accordingly [75]. Using inadequate sham interventions can result not only in unclear and deceptive outcomes but also in undervaluing these techniques and introducing bias against them [76].

After the first treatment, electro-acupuncture decreased brain activity in the right insular cortex and increased it in the left dorsolateral prefrontal cortex. In addition, the body mass index (BMI) was positively related to the right insular cortex activity at baseline. Electro-acupuncture decreased positive connectivity between the right insular cortex and the supplementary motor area and between the right insular cortex and right dorsolateral prefrontal cortex while also decreasing negative connectivity between the right insular cortex and the precuneus after the first treatment.

These changes suggest that electro-acupuncture has an acute impact on the patterns of communication within the brain, particularly involving the right insular cortex and, as discussed before, may be important in regulating interoception and inflammation.

On the other hand, the long-term effect of electro-acupuncture resulted in significant improvements in weight, BMI, emotional eating, and food addiction. In terms of brain activity, electro-acupuncture increased activity in the right ventrolateral prefrontal cortex, associated with tasks related to controlling impulses, suppressing unwanted memories, and managing various forms of inhibitory control [77,78,79]. It also plays a significant role in controlling impulses during physical activity [80] and in the process of making decisions and pursuing goal-directed behaviors [81].

In addition, post-treatment resting-state functional magnetic resonance imaging showed increased positive connectivity between right ventrolateral prefrontal cortex and bilateral orbitofrontal cortex. There was a negative correlation between the right ventrolateral prefrontal cortex and the right orbitofrontal cortex connectivity and BMI, suggesting enhanced dietary inhibition. Additionally, positive connectivity between the right ventrolateral prefrontal cortex and the left thalamus was observed, possibly contributing to decreased levels of depression and anxiety in obese subjects by reducing rumination, regulation of emotions, and cognitive control [80,82,83,84,85].

Overall, electro-acupuncture seems to have significant effects on brain activity and connectivity in overweight and obese patients, specifically suggesting that it can improve eating behaviors and aid in weight loss, which is also supported by other studies [86,87,88,89,90].

### 4.6. Final Remarks

The results of this review indicate that electro-acupuncture holds promise for effectively addressing a range of medical conditions, including carpal tunnel syndrome, fibromyalgia, Crohn’s disease, irritable bowel syndrome, and obesity. These positive effects are achieved through its influence on brain activity and connectivity. The specific mechanisms at play can vary depending on the condition and acupuncture methodology but often involve the modulation of pain perception, inflammation, and emotional regulation through alterations in brain connectivity and neurotransmitter levels. However, based on our results we may suggest several aspects:

- Localization of Effects:

Specific acupuncture points are associated with distinct brain regions and networks. For example, electro-acupuncture at ST25, CV6, and CV12 influences the homeostatic afferent processing network (insula and anterior MCC) for Crohn’s Disease, while electro-acupuncture at various points affects the anterior cingulate cortex, insular cortex, or the Default Mode Network depending on the condition.

- Somatotopic Representation:

The effects of electro-acupuncture seem to follow a somatotopic representation, meaning that the stimulation of specific body parts corresponds to specific areas in the brain. This is evident in the study on carpal tunnel syndrome, where local electro-acupuncture sites showed superior improvements, and in the fibromyalgia study, where the somatosensory component modulated the somatosensory–insular circuit.

- Condition-Specific Responses:

Different health conditions appear to elicit specific responses in the brain when treated with electro-acupuncture. For instance, electro-acupuncture for Crohn’s Disease involves modulation of networks related to afferent processing, while acupuncture for fibromyalgia focuses on the somatosensory–insular circuit.

- Functional Connectivity Changes:

Electro-acupuncture interventions can lead to changes in functional connectivity within brain circuits. The studies suggest that acupuncture may modulate brain function by influencing communication and inhibitory neurochemistry in specific circuits, such as the somatosensory–insular circuit for fibromyalgia or the prefrontal cortex for weight loss.

- Differential Effects of Acupuncture Components:

Different components of acupuncture interventions, such as electro-acupuncture or moxibustion, may have specific effects on brain mechanisms. For example, in the Crohn’s Disease study, electro-acupuncture mainly influenced the homeostatic afferent processing network, while moxibustion modulated the activity of the Default Mode Network.

While the overall quality of the included studies is generally good, there is room for improvement in their methodology, particularly in terms of sample size, which was generally low. Consequently, there is a need for more high-quality studies to validate and delve deeper into these mechanisms. Additionally, it is crucial to determine the optimal electro-acupuncture protocols for each specific medical condition.

Moreover, investigating the long-term effects and sustainability of the observed benefits is essential to gain a comprehensive understanding of electro-acupuncture’s therapeutic potential in these health conditions.

While our study provides valuable insights, it is important to acknowledge a major limitation arising from the inclusion of only five studies. This limitation constrains the overall scope and generalizability of our findings, emphasizing the need for cautious interpretation and recognition of potential variability in outcomes. To address this issue, we recommend that future analyses include a broader range of databases, potentially encompassing articles in Chinese. Furthermore, given the complexity of human brain functioning, our interpretation and discussion of the analyzed studies are constrained, highlighting the need for further research in this area.

Finally, there is a notable absence of studies comparing electro-acupuncture and classical acupuncture, raising questions about whether they would exhibit similar fMRI changes in the presence or absence of electrical stimulation.

## 5. Conclusions

The results of this review suggest that electro-acupuncture can have positive effects on various medical conditions, including carpal tunnel syndrome, fibromyalgia, Crohn’s disease, irritable bowel syndrome, and obesity, by influencing brain activity and connectivity. The findings suggest that electro-acupuncture can modulate neural pathways associated with pain perception, inflammation, emotional regulation, and homeostasis, contributing to symptom relief and improved clinical outcomes. More studies are warranted to confirm and further explore these mechanisms.

## Figures and Tables

**Table 1:** Characteristics of the included studies.

Authors—Year—Title—Journal	Sample	Condition	Groups	Technique	Duration and Frequency of Procedure	Assessments	fMRI Analysis/Task/Characteristics	Main Results	Quality Assessment
[19] Effect of electro-acupuncture and moxibustion on brain connectivity in patients with Crohn’s disease: A resting-state fMRI study *Frontiers in Human Neuroscience*	65 recruited, 38 completed the study	Crohn’s Disease	Electro-acupuncture group (n = 18) Moxibustion group (n = 20)	Electro-acupuncture (dense disperse, 2/100 Hz, 1–2 mA) and Moxa-cones (*Monkshood*, *Coptis chinensis*, *Radix aucklandiae*, *Carthamus tinctorius*, *Salvia* and *Angelica sinensis*) at bilateral ST25, CV6 and CV12	30 min. electro-acupuncture, 2 moxibustion cones, thrice a week for 12 weeks.	Crohn’s Disease Activity Index (CDAI), and Inflammatory Bowel Disease Questionnaire (IBDQ).	Resting-state fMRI/no task, performed 3 days before the treatment and 3 days after the end of treatment.	Electro-acupuncture treatment may modulate brain function mainly through the homeostatic afferent processing network (insula and anterior MCC). Moxibustion treatment may modulate the activity of the DMN (PCUN and IPC).	Reporting: 9 External validity: 3 Internal validity—bias: 6 Internal validity—confounding (selection bias): 3 Power: 0 Total Final score: 21 (Good)
[20] Comparison of electroacupuncture and mild-warm moxibustion on brain-gut function in patients with constipation-predominant irritable bowel syndrome: A randomized controlled trial *Chinese Journal of Integrative Medicine*	63 recruited, 60 completed the study	Irritable Bowel Disease	Electro-acupuncture group (n = 30). Moxibustion group (n = 30).	Electro-acupuncture and mild-warm moxibustion at bilateral ST25 and ST37	30 min, 6 times a week for 4 weeks.	Hamilton Anxiety Rating Scale (HAM-A) and Hamilton Depression Rating Scale (HDRS).	Before and after treatment, 7 patients in the electro-acupuncture group and 6 patients in the moxibustion group underwent fMRI examination voluntarily. 7 healthy volunteers from Jinhua Municipal Central Hospital staff and college interns, 22–45 years old, served as controls.	Electro-acupuncture significantly decreased activity in the anterior cingulate cortex area, and constipation-predominant irritable bowel syndrome patients increased activation of the insular cortex.	Reporting: 9 External validity: 3 Internal validity—bias: 6 Internal validity—confounding (selection bias): 5 Power: 1 Total Final score: 24 (Good)
[17] Rewiring the primary somatosensory cortex in carpal tunnel syndrome with acupuncture *Brain*	80 recruited, 56 completed the study	Carpel Tunnel Syndrome	Local acupuncture group (n = 28). Distal acupuncture group (n = 28). Sham acupuncture group (n = 23).	Local electro-acupuncture (2 Hz—continuous) at TW5 and PC7 and acupuncture at 3 chosen points amongst HT3, PC3, SI4, LI5, LI10 or LU5, all on the side of the most affected hand. Distal electro-acupuncture (2 Hz—continuous) at SP6 and LV4, and acupuncture at 3 chosen points amongst GB34, KD3 and SP5, all on the opposite side of the most affected hand. Sham electro-acupuncture (disconnected cables) at SH1 and SH2, and acupuncture points at SH3, all on the most affected arm, plus SH4 and SH5 on the opposite side leg.	20 min., twice a week for 8 weeks.	Boston Carpal Tunnel Syndrome Questionnaire (BCTQ), nerve conduction studies, and Diffusion tensor imaging (DTI).	fMRI scans were obtained at baseline and after the treatment. A vibrotactile stimulation over three digits (D2, D3, and D5) on the more affected hand was performed.	*Verum* and sham acupuncture reduced carpal tunnel syndrome symptoms. However, *verum* acupuncture was superior in improving both peripheral and brain neurophysiological outcomes. Improvements in functional S1 plasticity following acupuncture were related to long-term symptom relief. Acupuncture at local versus distal acupuncture sites might improve median nerve function at the wrist by somatotopically distinct S1-mediated neuroplasticity mechanisms.	Reporting: 8 External validity: 3 Internal validity—bias: 6 Internal validity—confounding (selection bias): 6 Power: 0 Total Final score: 23 (Good)
[18] Greater somatosensory afference with acupuncture increases primary somatosensory connectivity and alleviates fibromyalgia pain via insular γ-aminobutyric acid: A randomized neuroimaging trial. *Arthritis & Rheumatology*	76 recruited, 76 completed the study.	Fibromyalgia	Electro-acupuncture group (n = 33). Mock Laser Acupuncture group (n = 33).	Electro-acupuncture with somatosensory afference at right LI11 to LI4, left GB34 to SP6, and bilateral ST36, plus acupuncture at Du20, right ear Shen Men, and left LV3. Mock laser acupuncture, with no somatosensory afference, at the same points.	25 min., twice a week for 4 weeks.	Brief Pain Inventory (BPI), and proton magnetic resonance spectroscopy in the right anterior insula (H-MRS).	Resting-state fMRI/awake and eyes open images were collected at baseline and after the treatment.	Acupuncture’s somatosensory component specifically modulated functional communication and inhibitory neurochemistry in the somatosensory–insular circuit to improve fibromyalgia outcomes.	Reporting: 9 External validity: 3 Internal validity—bias: 5 Internal validity—confounding (selection bias): 3 Power: 0 Total Final score: 20 (Good)
[21] Acute and long-term effects of electroacupuncture alter frontal and insular cortex activity and functional connectivity during resting state *Psychiatry Research: Neuroimaging*	45 recruited, 32 completed the study	Overweight/Obesity	Electro-acupuncture group (n = 17). Sham acupuncture group (n = 15).	Electro-acupuncture (20 Hz—continuous) at CV12, CV10, ST25, SP15, ST21, ST24, ST26, ST27 and SP14.	30 min., thrice a week for one course of 6–8 weeks.	Self-rating depressive scale (SDS), Self rating anxiety scale (SAS), Yale Food Addiction Scale (YFAS), Dutch Eating Behavior Questionnaire (DEBQ), and Massachusetts Acupuncture Sensation Scale (MASS).	Acute effects: resting-state fMRI before and after the first acupuncture session. Long-term effects: resting-state fMRI before treatment and after treatment.	Electro-acupuncture and Sham decreased weight and body mass index. However, electro-acupuncture was superior at weight-loss. Electro-acupuncture-induced weight-loss was associated with the regulation of inhibitory-control in the prefrontal-cortex and brain regions, such as the insula, supplementary motor area, dorsolateral-prefrontal-cortex and dorsomedial-prefrontal-cortex, which are involved in gastric motility and satiety-control-related neural pathways in overweight/obesity.	Reporting: 9 External validity: 3 Internal validity—bias: 6 Internal validity—confounding (selection bias): 4 Power: 0 Total Final score: 22 (Good)

## Data Availability

Not applicable.
